# Effectiveness and Parental Acceptability of Social Networking Interventions for Promoting Seasonal Influenza Vaccination Among Young Children: Randomized Controlled Trial

**DOI:** 10.2196/16427

**Published:** 2020-02-28

**Authors:** Qiuyan Liao, Richard Fielding, Yee Tak Derek Cheung, Jinxiao Lian, Jiehu Yuan, Wendy Wing Tak Lam

**Affiliations:** 1 University of Hong Kong Hong Kong China (Hong Kong); 2 The Hong Kong Polytechnic University Hong Kong China (Hong Kong)

**Keywords:** influenza vaccination, social media, intervention, children

## Abstract

**Background:**

Seasonal influenza vaccination (SIV) coverage among young children remains low worldwide. Mobile social networking apps such as WhatsApp Messenger are promising tools for health interventions.

**Objective:**

This was a preliminary study to test the effectiveness and parental acceptability of a social networking intervention that sends weekly vaccination reminders and encourages exchange of SIV-related views and experiences among mothers via WhatsApp discussion groups for promoting childhood SIV. The second objective was to examine the effect of introducing time pressure on mothers’ decision making for childhood SIV for vaccination decision making. This was done using countdowns of the recommended vaccination timing.

**Methods:**

Mothers of child(ren) aged 6 to 72 months were randomly allocated to control or to one of two social networking intervention groups receiving vaccination reminders with (SNI+TP) or without (SNI–TP) a time pressure component via WhatsApp discussion groups at a ratio of 5:2:2. All participants first completed a baseline assessment. Both the SNI–TP and SNI+TP groups subsequently received weekly vaccination reminders from October to December 2017 and participated in WhatsApp discussions about SIV moderated by a health professional. All participants completed a follow-up assessment from April to May 2018.

**Results:**

A total of 84.9% (174/205), 71% (57/80), and 75% (60/80) who were allocated to the control, SNI–TP, and SNI+TP groups, respectively, completed the outcome assessment. The social networking intervention significantly promoted mothers’ self-efficacy for taking children for SIV (SNI–TP: odds ratio [OR] 2.69 [1.07-6.79]; SNI+TP: OR 2.50 [1.13-5.55]), but did not result in significantly improved children’s SIV uptake. Moreover, after adjusting for mothers’ working status, introducing additional time pressure reduced the overall SIV uptake in children of working mothers (OR 0.27 [0.10-0.77]) but significantly increased the SIV uptake among children of mothers without a full-time job (OR 6.53 [1.87-22.82]). Most participants’ WhatsApp posts were about sharing experience or views (226/434, 52.1%) of which 44.7% (101/226) were categorized as negative, such as their concerns over vaccine safety, side effects and effectiveness. Although participants shared predominantly negative experience or views about SIV at the beginning of the discussion, the moderator was able to encourage the discussion of more positive experience or views and more knowledge and information. Most intervention group participants indicated willingness to receive the same interventions (110/117, 94.0%) and recommend the interventions to other mothers (102/117, 87.2%) in future

**Conclusions:**

Online information support can effectively promote mothers’ self-efficacy for taking children for SIV but alone it may not sufficient to address maternal concerns over SIV to achieve a positive vaccination decision. However, the active involvement of health professionals in online discussions can shape positive discussions about vaccination. Time pressure on decision making interacts with maternal work status, facilitating vaccination uptake among mothers who may have more free time, but having the opposite effect among busier working mothers.

**Trial Registration:**

Hong Kong University Clinical Trials Registry HKUCTR-2250; https://tinyurl.com/vejv276

## Introduction

Seasonal influenza creates a substantial annual global disease burden. Young children are the most vulnerable age group [1,2], having higher viral loads and shedding the virus for a longer period than adults, making them important influenza viruses vectors to other household members [3]. Seasonal influenza vaccination (SIV) for children is therefore regarded as the most important measure to protect both children and the wider population [4] but uptake rates remain low in many countries [5-7]. In Hong Kong, families of children aged 6 months to 12 years receive a subsidy under the Childhood Influenza Vaccination Subsidy Scheme (CIVSS) to receive SIV from private-sector general practitioners. This policy removes financial barriers by making the vaccine completely free for the parents of target children, although some general practitioners demand an additional small administration fee. Despite the CIVSS, SIV uptake among young children in Hong Kong languishes around 30% [8,9]. Finding ways to improve SIV uptake thus remains crucial to reducing community influenza spread.

Sending vaccination reminders through mobile phone–based short message services (SMS) has been shown to promote vaccination uptake, including routine immunization and SIV in children [10-13] but reported effect sizes were small. A systematic review found that participants generally complained that mobile phone SMS reminders were limited by formats and character set [14]. The proliferation of mobile messaging apps and smartphone use has made mobile messaging functions more flexible compared with traditional SMS. In Hong Kong, WhatsApp messenger is used by over 80% of the population [15] through the high penetration of smartphone use [16]. In addition to providing flexible messaging functions like message structure, formats, and length, WhatsApp also permits social networking functions through creating multimember online discussion groups.

Existing vaccination reminders for promoting childhood SIV uptake have usually contained information on influenza infection risks and SIV benefits [13,17,18], key variables in cognitive theories of behavior change [19]. However, studies suggest that people inflate risk from vaccination relative to risk from natural infection possibly due to biased media coverage of vaccine risk [20] or omission bias, the tendency to believe that an error of omission is less serious than that from commission [21]. Therefore, merely providing information on influenza infection risks and influenza vaccination benefits may be insufficient to overcome concerns about vaccine-related risks, an important impediment to SIV uptake [8]. According to dual-processing models, information is not processed systematically and deliberatively but is widely influenced by heuristic cues that require less effort to reach a quick and efficient decision [22,23], particularly when participants feel uncertain and lack cognitive resources such as time and energy to make a decision. Previous studies suggest that parental decision making for children’s vaccination is extensively modified by knowing other parents’ vaccination decisions, indicating a strong social normative influence [8,24]. Others’ behavior provides important behavioral cues for social learning or imitation by indicating social approval, relieving safety concerns, and increasing confidence in specific choices [8,24]. Therefore, knowing that other parents take their child for SIV can encourage hesitant parents to do the same. This knowledge and experience sharing becomes more practical with messaging apps that enable social networking functions. However, few studies have examined the potential for social networking interventions to promote parental decisions about SIV for their children.

Studies in behavioral economics and neuroscience have suggested that introducing time pressure in decision making could increase decision makers’ reliance on heuristic cues for decision making, mainly through the mechanisms of acceleration (ie, switching to simpler strategies to speed up decision making) and selectivity (ie, automatically omitting certain information and favoring certain information) [25-27]. It is also suggested that while individuals can efficiently integrate different cues to reach an optimal decision under some time pressure, those under high time pressure can only use limited cues that are more salient for them (eg, heavily relying on negative cues) when making decisions [25,28]. Furthermore, time pressure may induce different affective states depending on individual capability to cope with the time limit and their cognitive load [26,27]. For individuals who perceive being able to make a decision within a time limit and have more cognitive resources to perform the decision task, time pressure could make them energetic and active in seeking risk reduction strategies. Otherwise, time pressure may induce stress that subsequently leads to more reliance on anecdotal cues rather than statistical information in decision making and thereby impairs their final decision [26,29]. Whether introducing time pressure can promote vaccination uptake or not may depend on how parents perceive the time pressure introduced in the vaccination decision. Hong Kong runs an annual influenza vaccination campaign (October to December) that recommends parents obtain SIV for their children aged 6 months to 12 years at least 2 weeks before the winter influenza season (January to March), allowing for sufficient time for the body to produce antibodies following vaccination. Therefore, the recommended optimal SIV window is from October until 2 weeks before the end of December annually, and as the winter influenza season approaches the optimal window diminishes, making vaccination decision making for parents naturally time-constrained. This provides an opportunity to test the effect of introducing time pressure to parental SIV decisions.

This preliminary study tested the effectiveness and parental acceptability of social networking interventions through the use of WhatsApp discussion groups for promoting children’s SIV uptake in Hong Kong. The specific objectives of this study were as follows:

- Examine the effectiveness of regularly delivering vaccination reminders and encouraging sharing positive SIV decisions and experiences through WhatsApp discussion groups in promoting target children’s SIV uptake
- Examine the effect of adding time pressure to parental SIV decisions (reminding parents about the remaining optimal SIV window)
- Conduct content analysis of WhatsApp discussion posts during the intervention period to examine how participants responded to childhood SIV and their interactions with the group moderator through WhatsApp discussions
- Examine acceptability to participants of using WhatsApp discussion groups as an example of social networking interventions for promoting child health

## Methods

### Overview

This study received ethical approval from the institutional review board of the University of Hong Kong/Hospital Authority Hong Kong West Cluster (reference number UW 17-003) and was registered with the Hong Kong University Clinical Trials Registry [HKUCTR-2250]. Participants were randomly allocated to either the control group, which received no intervention, or one of two social networking intervention groups that received weekly reminders to take their children for SIV via WhatsApp discussion groups with a time pressure component (SNI+TP) or without a time pressure component (SNI–TP) incorporated into the vaccination reminders. The intervention lasted for the 8 weeks of the Hong Kong government SIV campaign. Both intervention groups were also encouraged to share their positive vaccination decisions and experiences via their respective WhatsApp group with group members and a group moderator during the intervention period. A supermarket voucher valued at US $12.80 was given to every participant to improve response rate in the follow-up survey [30].

### Participants, Group Allocation, and Baseline Assessment

Since mothers in Hong Kong are the primary decision makers or significantly contribute to decision making with fathers for children’s immunization [8], this study only targeted mothers with at least one child aged 6 to 72 months to avoid confounding by gender effects. Other inclusion criteria were (1) Chinese communication fluency, (2) having a Hong Kong network-connected smartphone with internet access, and (3) having installed or being willing to install WhatsApp on their mobile phone. These inclusion criteria were intended to limit subjects to be primarily of Chinese ethnicity (who comprise approximately 93% of the Hong Kong population) to further minimize confounding by culture and language effects. Subjects were excluded if their eligible children had medical contraindications for immunization. Subjects were recruited before the 2017-2018 CIVSS campaign started and excluded if their target child(ren) had already received SIV for the 2017-2018 season. Eligible subjects were identified and recruited from previous samples of population-based random-dialed household telephone surveys and community outreach conducted by a commercial polling company previously used for successful population-based surveys [8,31]. All potential subjects were screened in a short telephone interview to confirm eligibility and obtain verbal consent for study participation. Each consenting subject was later called by a part-time telephone interviewer for an approximately 10-minute telephone baseline assessment interview. The baseline assessment collected data on participants’ and their children’s SIV history, sociodemographic characteristics, participants’ intention to take children for SIV during the 2017-2018 CIVSS campaign, and baseline risk perceptions regarding childhood influenza and the influenza vaccination. Before each telephone interview, the interviewer opened a sealed envelope which contained a random allocation sequence generated by computer to determine the subject’s group allocation. Subjects who were allocated to an intervention group were notified that they would be participating in a WhatsApp discussion group during the intervention period to receive weekly vaccination reminders and share their views and experiences about SIV with other mothers and a group moderator. This being a preliminary study to test the effectiveness of social networking interventions for promoting childhood SIV uptake, we aimed to recruit 200 subjects for the control and 80 subjects for each of the two intervention groups, allowing for a 30% dropout rate in each group, to detect an approximately 20% increase in vaccination uptake among the social networking intervention groups relative to the control with a power of 80% and 95% confidence interval. To balance confounding between study arms and control group size, blocked randomization [32] was used to allocate participants to one of the three arms, using a ratio of 5:2:2 for group allocation. Neither participants nor part-time interviewers performing subject recruitment and allocation could be blinded to subject allocation but the interviewers who conducted baseline assessment were blind to the intervention arm (with or without time pressure) participants occupied. The assessor of the primary outcome was blinded to all participant group allocation.

### Interventions

#### Vaccination Reminders

The vaccination reminder comprised three messages. Message 1 introduced the CIVSS and doctors’ recommendations for children’s SIV, message 2 addressed children’s risk of seasonal influenza and benefits and safety of SIV for children, and message 3 addressed the number of days remaining for the recommended vaccination timing (days remaining from the date when the vaccination reminder was sent out to the date 2 weeks before the winter influenza season). While the vaccination reminders for SNI–TP and SNI+TP contained message 1 and 2, message 3 (the time pressure component) was only included in the vaccination reminders for SNI+TP participants. All messages were constructed using information from the official websites of the Hong Kong Centre for Health Protection and World Health Organization and local published studies [33-35] and delivered in graphical format through WhatsApp. The messages contained mainly textual information, but graphical information was also incorporated to represent some key themes (eg, doctor’s recommendation, eligibility of CIVSS, and days remaining for optimal SIV window) and efficacy of SIV, aiming to improve audience comprehension and their attention and interest to read [36,37]. All messages were pretested using think-aloud interviews with 10 eligible mothers to ensure their readability via a mobile phone and comprehensibility without inducing negative feelings. Multimedia Appendix 1 gives the finalized messages in both the Chinese and English, but only the Chinese version was used in the intervention. Weekly vaccination reminders were assumed to be effective without increasing respondents’ information load with a preference for receiving vaccination reminders during afternoon [14]. Therefore, vaccination reminders were sent to the intervention groups midafternoon on different weekdays, weekly over the CIVSS campaign period from October to December 2017. The first vaccination reminder was delivered 2 weeks after the CIVSS started and the last one delivered on December 18, 2017, 2 weeks before the winter influenza season began. Overall, a total of 8 vaccination reminders were delivered to the intervention groups over the 8-week intervention period.

#### WhatsApp Discussion Groups

In addition to delivering weekly vaccination reminders, a WhatsApp discussion group was also set up to provide positive peer support for mothers to make better-informed SIV decisions regarding their children. To control group size and facilitate group discussion, participants allocated to the intervention groups were then randomly allocated to one of two SNI–TP and two SNI+TP WhatsApp discussion groups, each comprising approximately 40 mothers. In each WhatsApp discussion group, mothers could post their opinions and concerns about influenza and SIV and freely communicate with other mothers and the group moderator about their experiences of personal and child influenza vaccinations. The project moderator monitored and facilitated the group discussions on a daily basis following standardized guidelines (Multimedia Appendix 2). In addition to delivering weekly vaccination reminders via WhatsApp discussion groups, the moderator also sent one additional message on a weekly basis to enforce exchange of positive views and experience about SIV. The moderator also addressed any questions, concerns, or misunderstandings raised about influenza and influenza vaccination if these were not first addressed by other mothers within the groups. Posting content irrelevant to influenza and influenza vaccination was discouraged. Participation rules were set and delivered in the discussion groups immediately after the groups were created. Participants were informed that those violating the participation rules, such as using offensive statements and harassment, would be expelled from the discussion group. All members participating in the WhatsApp discussion groups were encouraged to use Chinese for communication. Voice messages were discouraged, and members were advised not to disclose names and other personal information to protect privacy. The WhatsApp discussion groups were closed by the project moderator 2 weeks after the last vaccination reminder was sent out.

### Outcome Assessment

**Figure 1:**
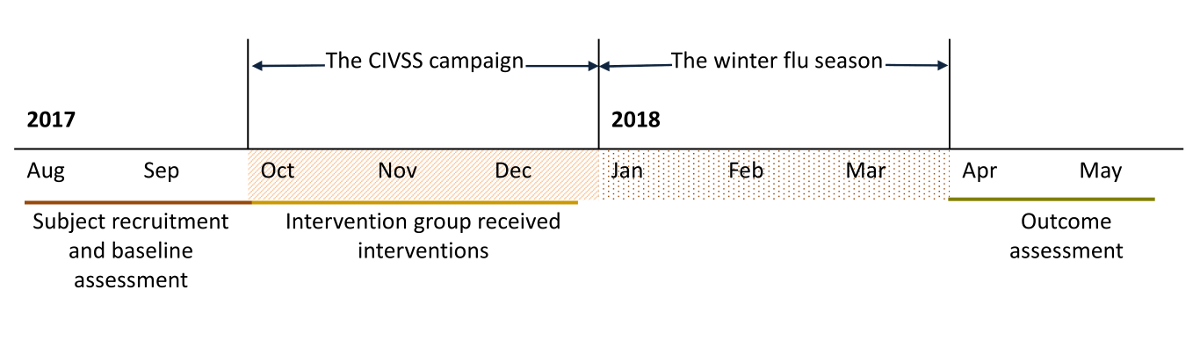
Timeline and study procedure. CIVSS: Childhood Influenza Vaccination Subsidy Scheme.

In April and May 2018 after the winter influenza season, all participants were again contacted to report information on their children’s SIV uptake before and during the 2017-2018 influenza season. For participants who had more than one child eligible for CIVSS, the vaccination status of each eligible child was recorded. Mother’s intention to take their children for SIV in the next 12 months was also recorded. Risk perceptions regarding seasonal influenza and SIV for children were assessed again to examine whether any changes in perceptions occurred after the interventions. Participants’ opinions about the interventions and their willingness to receive vaccination reminders via WhatsApp in the future were asked to assess the acceptability of the interventions. In addition, a total of 20 participants from the intervention groups were contacted from May to July 2018 for in-depth interviews to explore their opinions about interventions and the acceptability of using WhatsApp for promoting children’s health. Figure 1 illustrates the study procedure and timing.

### Data Analysis

Pearson chi-square tests were first conducted to compare participants’ demographics, baseline perceptions, history of influenza vaccination, and their target child’s characteristics by intervention arm to assess randomization and by follow-up status to assess selection bias.

#### Assessment of Primary Outcomes

Children’s SIV uptake rate in 2017-2018 was calculated for each group and compared between groups using the Pearson chi-square test. Both the SIV uptake of all target children aged between 6 to 72 months and that of the youngest target child’s SIV were compared across groups, because among families with more than one target child, the youngest one tends to be not vaccinated [9]. The intervention effect on children’s SIV uptake was also examined by stratifying the analyses by participants’ educational attainment, work status, and household income to identify potential sociodemographic effect modifiers previously reported to be associated with parental acceptance of influenza vaccination for their children [38-40].

To further assess the effects of the interventions on vaccination uptake, a generalized estimating equation (GEE) logistic regression model was conducted to examine the following questions: (1) Did SIV outcome differ by intervention arm (intervention effect)? (2) Did SIV outcome change from baseline to follow-up (time effect)? (3) Did change of SIV outcome by time differ by intervention arm (intervention × time interaction)? GEE can accommodate cases with missing outcome measures at some time points (cases with outcome measure at one time point will be counted) and the correlation between the outcome measures at different time points (ie, the baseline and follow-up SIV uptake) [41]. Potential effect modifiers (eg, participants’ demographics) identified in the univariate analysis would be additionally included in the GEE to test its interaction effects with both the time and intervention on the outcome.

In the GEE analysis, participants’ youngest target child’s SIV status during the follow-up period was used as the outcome. Since the final SIV uptake of the target child(ren) of participants who dropped out at follow-up was unavailable, intention-to-treat analysis was used as a conservative and sensitivity analysis by treating the lost outcomes as not vaccinated over the specific CIVSS campaign to compare with the complete case analysis.

#### Assessment of the Secondary Outcomes

Excepting for effects on children’s SIV uptake, intervention effects on parental perceptions regarding influenza and SIV by intervention arm were also assessed using chi-square and similar GEE logistic regression modeling. All WhatsApp group posts were archived by the project moderator immediately before the WhatsApp discussion groups were closed.

The mean number of posts per participant was calculated while the distributions of participants’ frequency of posting across discussion groups were compared using Kruskal-Wallis equality-of-populations rank tests. All discussion posts were examined to further explore participants’ responses to the vaccination reminders, their perceptions and attitudes regarding influenza and influenza vaccination, and how they interacted with peers and the group moderator during the communication process.

All posts were analyzed and coded by two researchers independently using content analysis. Each post was coded for the following categories: role (moderator or participant), format (text, picture, emoji, or hyperlink), cybersupport (eg, sharing views or experience and emotional exchange) and discussion topics (eg, vaccine effectiveness, vaccine safety, and side effects). More than one code could be assigned to each post. A coding scheme for cybersupport and discussion topics was drafted and developed by the first author based on literature on online psychosocial support [42,43] and parental decision making for childhood influenza vaccination and vaccination attitudes [8,24] and refined throughout data analysis and the discussion of the research team.

The refined coding scheme was then used in NVivo 12.0 (QSR International Pty) by the first author and a trained research assistant to independently code all the posts again. The interrater agreement between the two coders was assessed; the Cohen kappa was less than 0.6, indicating low agreement, which was then resolved by joint discussion between the two coders.

How the moderator’s involvement in the WhatsApp discussion could change the discussion direction about SIV among participants was also analyzed by plotting the time sequence of cybersupport behaviors of participants and the moderator in each discussion group. Parental acceptability of the intervention was first assessed by describing participants’ opinions about the interventions and their willingness to receive vaccination reminders via WhatsApp in the future. In addition, thematic coding was conducted to identify themes and categories relating to parental acceptability of the interventions and using WhatsApp Messenger for child health promotion emerging from the in-depth interviews. All quantitative data were analyzed using Stata 15.1 (StataCorp LLC) while the textual data were analyzed using NVivo 12.0.

## Results

### Participants

A total of 365 mothers in the control, SNI–TP, and SNI+TP groups completed the baseline assessment, of whom 85.9% (174/205), 71% (57/80), and 75% (60/80), respectively, completed the outcome assessment. Two participants of the SNI/–TP left the group in the first week of the intervention without giving any reasons and another 2 participants of the SIN/–TP left in the fifth week of the intervention for violating participation rules with offensive statements when arguing over SIV for their children. Participants of the intervention groups were more likely to drop out from the outcome assessment than were the control (χ^2^_22_=8.0, *P*=.02), but those who completed the baseline assessment and outcome assessment did not differ by intervention condition in terms of their demographics, their target child’s characteristics, past SIV uptake, baseline SIV perceptions, and intention to take child for SIV (Table A of Multimedia Appendix 3). Almost all participants used WhatsApp on a daily basis across the intervention arm (Table A of Multimedia Appendix 3).

### Intervention Effects on the Target Child’s Seasonal Influenza Vaccination Uptake

The youngest target child SIV uptake rates were 37.9% (66/174), 33% (19/57), and 38% (23/60) in the control, SIN–TP, and SNI+TP groups, respectively. Chi-square tests indicated that the interventions did not have significant effects on either the youngest target child’s SIV uptake or all target child(ren)’s SIV uptake (Table 1). It also shows that the youngest child’s SIV uptake appeared to be greater in the SNI+TP group for participants who did not have a full-time job (χ^2^_22_=5.31, *P*=.07), suggesting that participants’ work status may be a potential effect modifier (Table 1 and Multimedia Appendix 4).

GEE analysis was conducted to further take into account the time effect (SIV uptake rate changed from the baseline to the follow-up) and its interaction with the intervention condition as well as its interaction with both intervention condition and participants’ work status. Results showed that the youngest target child’s SIV uptake rate significantly increased from the baseline to the follow-up (OR 3.13, 95% CI 2.14-4.57) in all groups, but such increase was shown to be significantly less in the SNI+TP group than the control (OR 0.27, 95% CI 0.10-0.77) after adjusting for participants’ work status. Participants’ work status significantly interacted with both the time and intervention effects, with the target child’s follow-up SIV uptake increased significantly more among participants who did not have a full-time job than the control (OR 6.53, 95% CI 1.87-22.82; Table 2). The intention-to-treat analysis yielded a similar conclusion (data not shown).

**Table 1:** Seasonal influenza vaccination uptake rates among target children at the follow-up by intervention condition.

Characteristic			Control (n=174), % (95% CI)	SNI–TP^a^ (n=57), rate (95% CI)	SNI+TP^b^ (n=60), rate (95% CI)	*P* value^c^
SIV^d^, youngest target child			37.9 (30.7-45.6)	33.3 (21.4-47.1)	38.3 (26.0-51.8)	.80
**SIV uptake, all target children**						.78
	All		37.4 (30.2-45.0)	33.3 (21.4-47.1)	38.3 (26.1-51.8)	
	Partial		4.0 (1.6-8.1)	21.7 (0-9.4)	21.7 (0-8.9)	
**Demographics, youngest target child**						
	**Educational attainment**					
		Secondary or below	37.1 (25.9-49.5)	33.3 (15.6-55.3)	46.7 (28.3-65.7)	.56
		Tertiary or above	38.5 (29.1-48.5)	33.3 (18.0-51.8)	30.0 (14.7-49.4)	.66
	**Household income (HK$ [US $0.13])**					
		40,000 or below	37.0 (27.1-48.0)	20.0 (6.8-40.7)	36.0 (18.0-57.5)	.27
		More than 40,000	38.8 (28.4-50.0)	43.7 (26.4-62.3)	40.0 (23.9-57.9)	.89
	**Work status**					
		Full-time	37.6 (27.8-48.3)	31.8 (13.9-54.9)	16.7 (5.6-34.7)	.10
		Part-time/unemployed	38.3 (27.7-49.7)	34.3 (19.1-52.2)	60.0 (40.6-77.3)	.07

^a^SNI–TP: social networking intervention group who received weekly vaccination reminders without time pressure component.

^b^SNI+TP: social networking intervention group who received weekly vaccination reminders with time pressure component.

^c^*P* values were calculated using Pearson chi-square test.

^d^SIV: seasonal influenza vaccination.

**Table 2:** Assessment of the intervention effects on child’s influenza vaccination uptake using generalized estimating equation logistic regression.

Independent variables		Beta (SE^a^)	Odds ratio (95% CI)	*P* value
**Intervention**				
	SNI–TP^b^ (vs control)	–0.20 (0.38)	0.82 (0.38-1.71)	.59
	SNI+TP^c^ (vs control)	0.24 (0.34)	1.27 (0.65-2.47)	.65
Time effect: follow-up versus baseline		1.14 (0.19)	3.13 (2.14-4.57)	<.001
Time × SNI–TP		–0.002 (0.51)	1.00 (0.36-2.73)	.95
Time × SNI+TP		–1.29 (0.53)	0.27 (0.10-0.77)	.01
Work status (part-time/unemployed vs full-time)		0.14 (0.24)	1.15 (0.72-1.83)	.56
Time × SNI–TP × part-time/unemployed		–0.03 (0.60)	0.97 (0.30-3.17)	.96
Time × SNI+TP × part-time/unemployed		1.88 (0.64)	6.53 (1.87-22.82)	.003

^a^SE: standard error.

^b^SNI–TP: social networking intervention group who received weekly vaccination reminders without time pressure component.

^c^SNI+TP: social networking intervention group who received weekly vaccination reminders with time pressure component.

### Intervention Effects on Participants’ Perceptions of Influenza and Seasonal Influenza Vaccination

**Figure 2:**
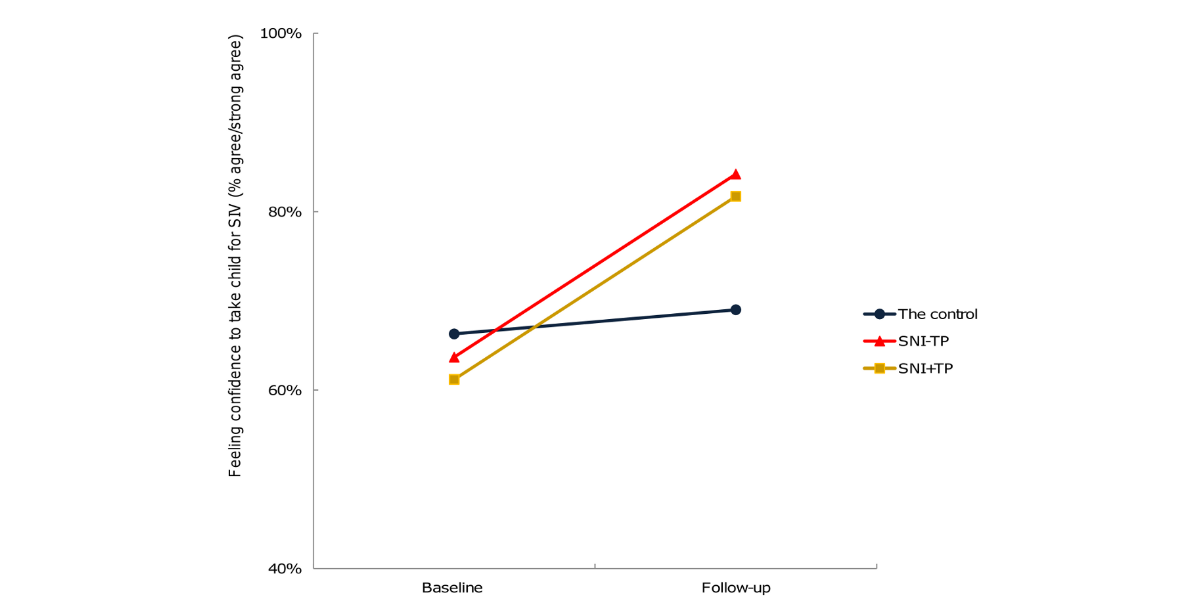
Change in participants' perceived self-efficacy for taking child for seasonal influenza vaccination by intervention condition. SNI–TP: group that received weekly reminders to take their children for SIV via WhatsApp discussion groups without a time pressure component; SNI+TP: group that received weekly reminders to take their children for SIV via WhatsApp discussion groups with a time pressure component; SIV: seasonal influenza vaccination.

GEE analysis was also conducted to examine whether change in participants’ SIV perceptions from the baseline to the follow-up differed by intervention condition. Results showed that there were significant intervention effects on the change of participants’ perceived self-efficacy in taking children for SIV, with participants of the SNI–TP (OR 2.69, 95% CI 1.07-6.79) and SNI+TP (OR 2.50, 95% CI 1.13-5.55) groups reporting more increase in confidence in taking their children for SIV than did the control participants (Figure 2 and Table B of Multimedia Appendix 3).

### Content Analysis of WhatsApp Discussion Group Posts

From four WhatsApp discussion groups including two SNI–TP groups and two SNI+TP groups, after excluding posts irrelevant to influenza, vaccination, or children’s health (2.7% [12/446] of the total posts), 434 posts from participants were retrieved over 8 weeks, on average 13.6 posts per group per week. Overall, 58.1% (93/160) of the participants who joined the WhatsApp discussion groups participated in the online discussion, on average 3.08 posts (SD 5.90) per participant (Table C of Multimedia Appendix 3). There was no significant difference in the distribution of number of posts made by participants across the four discussion groups (χ^2^_23_=2.72, *P*=.44). Of the 434 relevant participant posts, 119 (45.8%) were made after office hours, but all posts seeking information or opinions were addressed within 24 hours. The project moderator delivered 203 posts in total, apart from weekly vaccination reminders, for the four discussion groups, on average 6.34 posts per group per week. Most posts were textual but graphical information, hyperlinks of news articles, and emoji were also used (Table C of Multimedia Appendix 3). All relevant participant and moderator posts excluding the weekly vaccination reminders were coded for themes and categories relevant to cybersupport and discussion topics.

#### Cybersupport

Of 434 participant posts, 226 (52.1%) were coded as sharing experience or views, 119 (27.4%) as seeking information or opinions, 106 (24.4%) as sharing knowledge or information, and 66 (15.2%) as emotional exchange (Table 3). The experience or views shared by participants were categorized as being negative (101/226, 44.7%) or positive (87/226, 38.5%) based on whether the experience or views had a positive or negative effect for motivating SIV uptake [19]. Posts categorized as seeking information or opinions were often asking the moderator questions but some also involved sharing experience or views (Table 3). Sharing knowledge or information is distinguished from sharing experience or views because the former mainly refers to providing information support for vaccination decision. Emotional exchange reflected, for example, participants’ expression of appreciation after receiving information from others, worry or concerns (over vaccine safety), feeling doubt or confusion due to different opinions, and difficulty in making vaccination decisions, mostly comprising the use of emoji icons. Of 203 moderator posts, most were sharing knowledge or information followed by encouraging information, experience sharing, and encouraging vaccination planning (Table 3).

**Table 3:** Quotes about cybersupport from the WhatsApp discussion groups.

Cybersupport and number of posts			Quotation
**Participant posts (n=434)**			
	**Sharing experience or views (226/434, 52.1%)**		
		Negative (101/226, 44.7%)	I also do not take my child for flu vaccination because it can be worse if he got a fever after taking vaccination. I have to work and don’t want to take leave to take care of him (after vaccination).
		Positive (87/226, 38.5%)	I took my 3-year-old son for flu vaccination today. He also took the flu vaccination when he was two years old. I think it is necessary. Now, we cannot overlook the risk of influenza. In addition, the viruses change more and more easily. It is necessary to give children the prevention. We should take our children for the vaccination even if there is no subsidy from government.
		Neutral/Mixed (39/226, 17.3%)	I’m indecisive...Don’t know whether I should take my child for the vaccination.
	Seeking information or opinions (119/434, 27.4%)		I want to ask: it is my baby’s first flu vaccination. What can be the maximum time interval between the two doses of flu vaccine? Is it true that one has to take flu vaccination every year once he/she takes the first flu vaccination?
	Sharing knowledge or information (106/434, 24.4%)		There are still some quadrivalent influenza vaccines at Dr XXX in Yuen Long. The vaccination is free there. You may call the clinic for more information if your child hasn’t received the vaccine. They provide flu vaccination during weekends.
	Emotional exchange (66/434, 15.2%)		Thank you for sharing the information. I’m considering (whether to take my child for flu vaccination (or not) feeling uncertain.
**Moderator posts (n=203)**			
	Sharing knowledge or information (144/203, 70.9%)		All children aged 6 months to 8 years who have never received flu vaccine or those who just received one dose of flu vaccine at their first-time vaccination should receive two doses of flu vaccine.
	Encouraging information and experience sharing (42/203, 20.7%)		Mothers who have taken your child for influenza vaccination can share your experience!
	Encouraging vaccination planning (21/203, 10.3%)		According to our survey, most parents indicated intention to take their children for flu vaccination. Mothers who have such intention are encouraged to plan your child’s vaccination early.
	Encouraging information seeking (20/203, 9.9%)		We understand that the people in the public have different opinions about influenza vaccination. We should carefully evaluate the evidence and the sources of the information. Surely, as a parent, you are the main decision maker for your child’s flu vaccination. You are encouraged to discuss with your family doctor if necessary.
	Sharing experience or views (14/203, 6.9%)		I remember, at the second time when I took my daughter to take flu vaccination, she cried out as soon as she saw the nurse. But, we can’t care too much about her crying because the vaccination can protect her from diseases.

#### Discussion Topics

The main discussion topics among participants’ posts are shown in Table 4. The most common participant discussion topics were vaccination decisions followed by vaccination clinic and cost, vaccine safety and side effects, and vaccine effectiveness (Table 4). Most participant posts on vaccination decisions met criteria for being categorized as positive vaccination decision (intending to take/planning to take/have taken children for SIV during the intervention period) (69/134, 51.9%) while the remaining were coded as being negative or hesitant about seeking opinions for vaccination decision. Most participant posts on vaccination clinic and cost comprised information shared by participants in support of SIV vaccination (48/63, 76.2%) with the remainder about seeking information on vaccination clinic or cost. Participants raised a number of concerns over vaccine safety, side effects, and vaccine effectiveness or had doubtful or negative vaccination attitudes. These concerns or views about SIV seem to mostly reflect beliefs that SIV could weaken immunity, distrust about how the vaccine strain was estimated every year, and a perception that vaccination is not a natural process. Vaccination experience is distinguished from vaccination decision or plan because it mainly refers to participants’ feeling about the vaccination process (eg, injection pain) or after vaccination (more or fewer illnesses). Most participant posts on medical eligibility of SIV and first-time influenza vaccination belonged to seeking information or opinions.

**Table 4:** Quotes from main discussion topics of participant posts (n=434).

Discussion topics and number of posts		Quotation
**Vaccination decision (134/434, 30.9%)**		
	Positive (69/134, 51.5%)	I will take my child for flu vaccination. I also have booked an appointment to take my son for flu vaccination.
	Negative (40/134, 29.9%)	I won’t take my child for flu vaccination because there is still some negative news.
	Being hesitant or seeking opinions for vaccination decision (25/134, 18.7%)	I am considering (whether to take my child for flu vaccination). Then, should I take my child for flu vaccination?
**Vaccination clinic and cost (63/434, 14.5%)**		
	Sharing information (48/63, 76%)	Dr XXX at Kwai Fong, trivalent vaccine is free and quadrivalent vaccine cost HK$60. My child just took the vaccination yesterday, and they still have some available vaccines.
	Seeking information (15/63, 24%)	Which clinics provide free flu vaccination (for children)?
**Vaccine safety and side effects (62/434, 14.3%)**		
	Concerns over vaccine safety and side effects (40/62, 65%)	Is it true that one needs to take influenza vaccination every year once he/she receives the first flu vaccination and that all family members should receive influenza vaccination once one member of the family receives the flu vaccination (otherwise it can be worse)?
	Being mixed or neutral/purely seeking information about vaccine safety and side effects (16/62, 26%)	Different children may have different reactions to the flu vaccination. What can be the side effects of flu vaccination?
	Sharing information for clarifying vaccine safety and side effects (6/62, 10%)	It is misinformation that vaccination can cause autism. This rumor has been dismissed many years before.
**Vaccine effectiveness (52/434, 12.0%)**		
	Concerns over vaccine effectiveness (26/51, 51%)	Now there are too many viruses/bacteria, and they change very quickly. This time, we take the flu vaccination against this virus but later another new virus emerges. How can we ensure that the vaccination is effective? It depends on how accurate their guess on the vaccine strain is every year. If their guess is wrong, the flu shot is a meaningless suffer. If one can still get sick even after taking the vaccination, why should he suffer from an injection?
	Sharing information for clarifying vaccine effectiveness (16/51, 31%)	Although there is mismatch, the vaccine is still effective for preventing influenza H1N1 or influenza B viruses. It (flu vaccination) is an additional protection for our children.
	Being mixed or neutral/purely seeking information about vaccine effectiveness (15/51, 29%)	Is it true that one can still get a cold even after taking the vaccination but can protect against influenza? Can influenza vaccination protect one against serious complications due to influenza?
Medical eligibility for seasonal influenza vaccination (40/434, 9.2%)		I thought to take my daughter for flu vaccination today but she has a running nose and some cough. Is it OK for her to take flu vaccination?
**Vaccination experience (33/434, 7.6%)**		
	Positive (16/33, 49%)	My child has taken the flu vaccination and he still feels very good now.
	Negative (12/33, 36%)	My elder daughter took the flu vaccination once but got more and severe sicknesses that year. Since then, she has never taken flu vaccination...
	Mixed or uncertain (5/33, 15%)	My two sons have taken the flu vaccination. One is 3 years old. He was given injection in the hip and he said no pain. Another is 7 years old. He was given injection in the arm. He said it was very painful and the pain lasted for 2 days.
Doubtful or negative vaccination attitudes (26/434, 6.0%)		Vaccination is to inject germs into the body. Is it necessary to take flu vaccination if my child is always healthy? Too many vaccinations are not good for children.
First-time influenza vaccination (20/434, 4.6%)		I would like to ask: it is my baby’s first flu vaccination. The doctor said he needed two doses of vaccines. Then what’s the maximum time interval between the two vaccinations?

The main knowledge and information shared by the moderator was about vaccine effectiveness (30/144, 20.8%), vaccination clinic and cost (27/144, 18.8%), vaccine safety and side effects (25/144, 17.4%), medical eligibility for SIV (18/144, 12.5%), and first-time influenza vaccination (15/144, 10.4%). The moderator also provided social cues related to vaccination (eg, doctors’ recommendation, other mothers’ decisions to take their child for SIV, and vaccination statistics) to motivate vaccination decision or planning (23/144, 16.0%).

### Interactions Between Participants and the Moderator During Online Discussion

**Figure 3:**
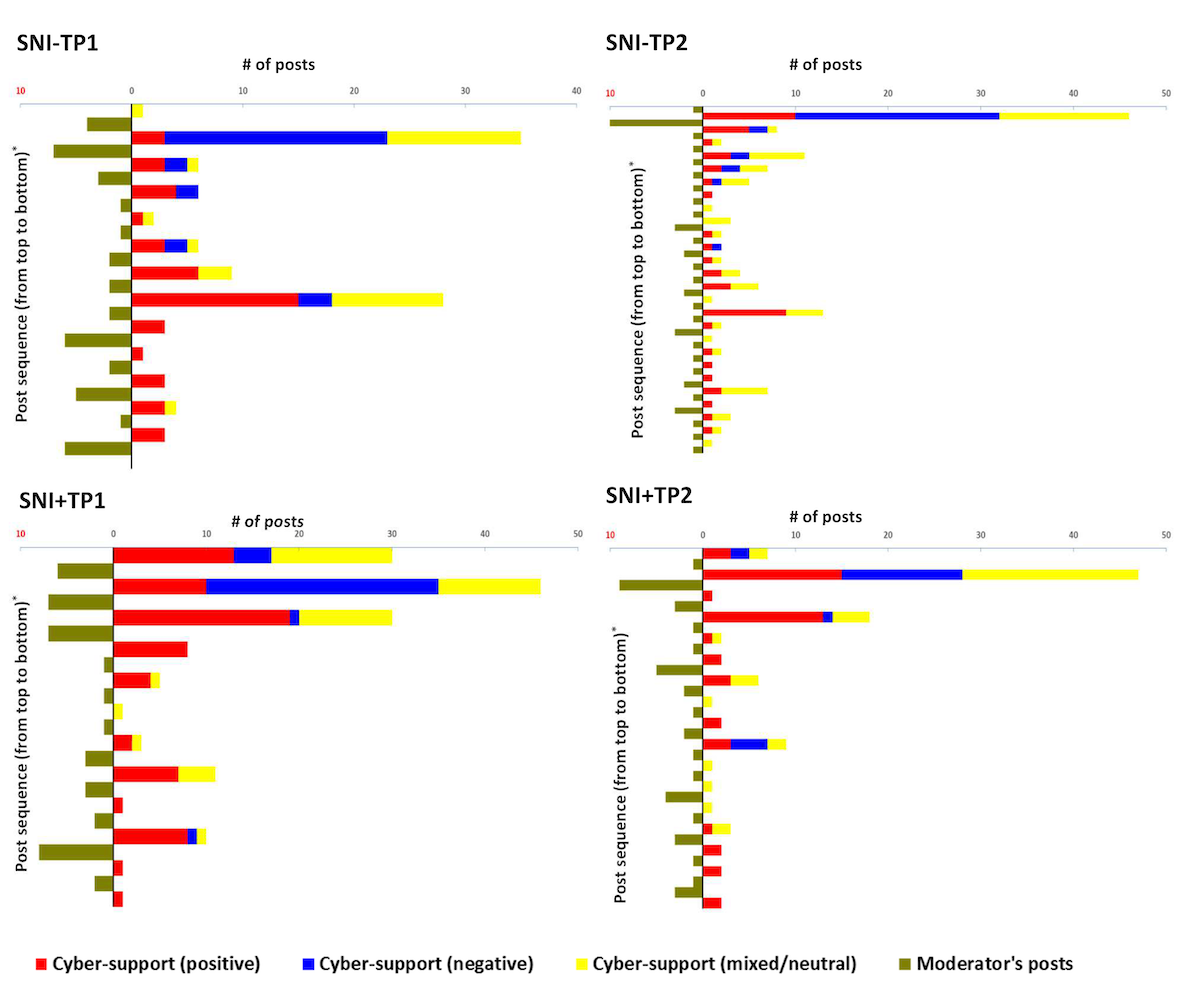
Change of cybersupport behaviors among participants by time and moderator’s involvement. SNI–TP1 and SNI–TP2: groups that received weekly reminders to take their children for SIV via WhatsApp discussion groups without a time pressure component; SNI+TP1 and SNI+TP2: groups that received weekly reminders to take their children for SIV via WhatsApp discussion groups with a time pressure component; SIV: seasonal influenza vaccination.

To illustrate the change of participant cybersupport behaviors as the moderator became involved in the online discussion, participant cybersupport behaviors were categorized into three types based on their potential effects on SIV uptake: positive cybersupport behaviors comprising sharing positive experience or views, sharing knowledge or information and positive emotional exchange; negative cybersupport behaviors comprising sharing negative experience or views and negative emotional exchange; and mixed or neutral cybersupport behaviors comprising sharing mixed or neutral experience and views, seeking information or opinions, and other emotional exchange. Figure 3 shows that although participants mainly shared their negative experiences, views, or emotions (blue bars) regarding SIV at the beginning of the online discussion, with the moderator’s involvement throughout the discussion, the numbers of posts sharing positive experience or views, sharing knowledge or information, and positive emotional exchange (red bars) increased. However, the discussion dynamic also indicates a less active participation in the discussion among the participants as the discussion proceeded.

### Parental Acceptability of the Intervention

Of the 117 participants of the intervention groups who completed the outcome assessment, 115 (98.3%) reported reading the discussion posts at least several times a week during the intervention period and 105 (89.7%) had read more than one-half of all discussion posts. Over 80% (95/117, 81.2%) indicated no concern over participating in the WhatsApp discussion groups. Of those expressing concerns, the most common concern was receiving misinformation or irrelevant information. Most (93/117, 79.4%) agreed that the information from the discussion groups could improve understanding about SIV. Around 60% (70/117, 59.8%) agreed that the information was useful but 20.0% (23/117) reported the information was insufficient for SIV decision making. Overall, 94.0% (110/117) were willing to accept the same intervention in the future, 84.6% (99/117) would recommend the intervention to other mothers, and 87.2% (102/117) were satisfied with the moderator’s information.

Post hoc qualitative interviews with 20 participants of the intervention groups were analyzed to clarify participants’ in-depth opinions about the interventions (Table D of Multimedia Appendix 3). One main theme emerging from the interviews addressed perceptions of information from the moderator comprising information attributes, benefit of information provision, and lack of interest in information. Most participants emphasized the positive attributes of the moderator’s information but a few complained that the reminders were too repetitive and that the moderator’s responses lacked details. Two participants mentioned the unbalanced presentations of the pros and cons of influenza vaccination, giving an impression of hard sell. Benefits of information provision comprise knowledge acquisition, moving to a contemplation stage, promoting motivation for taking vaccination, and reminding of vaccination planning. The second theme is perceived advantages of using WhatsApp for promoting child health comprising convenience in information accessibility, better information quality, and enhanced interaction with a health professional. Few concerns over using WhatsApp for health promotion were raised, mainly regarding receiving unwanted advertising. On perceptions of the time pressure component, most reported feeling pressured into making a rapid decision, either a positive or negative one, but others ignored or failed to notice the shrinking optimal window of time. Contributors’ reasons for not participating in the online discussion included perceived low confidence about giving information, avoiding arguments, and perceived low information need.

## Discussion

### Principal Findings

This social networking intervention, involving sending weekly vaccination reminders and encouraging exchanges of positive experiences and information among participants via WhatsApp discussion groups during an influenza vaccination campaign, did not significantly enhance children’s SIV uptake. Two main reasons may explain why a significant effect of sending regular vaccination reminders was not identified. First, compared with previous studies that used vaccination reminders to promote routine childhood immunization [11,12], our study focused on promoting an optional vaccine, childhood SIV; parents have more risk-related concerns about optional vaccines [24]. Our qualitative data indicated that although the positive attributes of information from the moderator were appreciated by most participants, the information provided mainly improved knowledge, motivated contemplation, and increased vaccination motivation. For participants who had already made the decision to take their children for SIV before joining in the discussion group, the information may have prompted vaccination planning or been used as cues for taking action. For participants who had antivaccination attitudes or were hesitant to take SIV, the information was insufficient to change the psychological roots of the antivaccination attitudes [44] or remove concerns over vaccine risk and thereby cannot support a final decision for or action on children’s SIV. Second, compared with studies that found a positive effect of sending regular vaccination reminders for promoting influenza vaccination [10,13,17,18], vaccination reminders were delivered by a health professional researcher (the moderator) rather than a general practitioner on the primary care team who had access to the target children’s medical records. Therefore, although information from the moderator was perceived by participants to be trustworthy, it may have been perceived as less relevant to children’s health care compared with information received directly from a general practitioner and thereby had less impact on parental SIV decision making. However, except for children with chronic conditions, most parents and their children may not frequently interact with a primary care team. Therefore, although this reflects one potential weakness of our study, it may be more representative of a real public health scenario for promoting childhood SIV. Other studies suggest that even the health care providers’ position on vaccine safety is being increasingly questioned by parents [45,46]. Health care providers need to communicate carefully with vaccine-hesitant parents. Our study indicates that the health professional’s active participation and involvement in vaccination discussions can create a more positive online experience. The internet has become probably the main information source shaping negative parental attitudes around childhood immunization [47-49]. Active communication from health professionals may be sufficiently effective to combat vaccine hesitancy compared with attempts to control online media misinformation [50,51].

Despite not increasing SIV uptake among the target children, the social networking intervention was significantly effective for promoting mothers’ self-efficacy in taking their children for SIV. This is possibly due to the frequent posts of information about the vaccination clinics and cost that were shared by both moderator and participants through the online discussion. Previous studies also have found that online information support significantly increased parents’ perceived self-efficacy in other child health care practices [52-55] and that peer experience-based information may be more likely to meet their information needs [56,57]. As parents’ perceived self-efficacy for taking children for SIV is a significant predictor for children’s SIV uptake [8], this is likely to facilitate future childhood SIV uptake. However, the discrepancy between the enhanced parental self-efficacy in taking child for SIV and the unchanged SIV uptake indicates that the direct effect of perceived self-efficacy on vaccination uptake is weak [8]. Enhanced self-efficacy should combine with positive vaccination attitudes to promote positive vaccination decision. However, the moderator was found to be the main source of knowledge and information about vaccine safety, side effects, and effectiveness, while participants generally felt a lack of confidence in sharing their personal knowledge, particularly when there was a health professional (the moderator) in the group. Because experience-based knowledge and information from peers may be more powerful and persuasive for changing parents’ attitudes [56,57], future studies should focus on how to encourage peers to share positive experience-based knowledge and information about vaccine safety, side effects, and effectiveness for promoting childhood vaccination.

Including an additional time pressure did not significantly enhance childhood SIV uptake. However, subgroup analysis showed that children’s SIV uptake significantly increased among mothers without a full-time job while declining slightly among mothers with a full-time job when the time pressure intervention was included. The qualitative data indicated that time pressure pushed participants to make a rapid decision, but those decisions can be either positive or negative. Unemployed and part-time-employed mothers may have more cognitive resource to deliberate the pros and cons of influenza vaccination and perceive that they have the ability to make the decision within time limit. Therefore, under some time pressure, they may become more active in searching information to reduce the risk of influenza and efficiently integrate different cues to reach a positive vaccination decision. In comparison, working mothers face more pressure from work for childcare [40] and thereby tend to have more concerns over disruptive vaccination side effects (proximal cost) than the risk of influenza (distal cost). Working mothers may also place more weight on the value of time taken from work to seek vaccination for their children [40] and thereby the negative cues that favor inaction (not vaccinate the child) may become more salient for them. As working mothers may have fewer cognitive resources to decide whether to take their children for SIV, the time pressure is likely to induce stress in decision making. Therefore, time pressure may enforce the influence of negative cues (eg, side effects of influenza vaccination) on the vaccination decisions among working mothers.

The content analysis of the WhatsApp discussion identified several maternal concerns and misperceptions about SIV. Two common concerns about vaccine side effects were that SIV was needed annually once initiated and that all family members should be vaccinated if one member was vaccinated. These concerns seem linking to beliefs that SIV weakens immunity. This may be a misinterpretation of current recommendations for annual SIV vaccination of all family members which should be addressed in future SIV risk communications. Similarly, vaccine effectiveness was an issue because SIV does not ensure 100% protection and is worse where the SIV strain does not match the actual circulating strain. SIV was perceived to be useless or wasteful by participants. This may also link to a common distrust about how vaccine strains are predicted by the vaccine scientific committee. Future risk communication should clarify the accuracy of existing prediction for the main influenza vaccine strain and the effectiveness of SIV in protecting against not only risk of getting influenza but also complications of influenza illnesses, and even when strains are not matched, SIV can still offer some cross-immunity. Some participants refused SIV due to their belief that vaccination is not a natural process. Future risk communication should give a clear explanation about the mechanism of influenza vaccination, which is a quasi-natural process, by emphasizing similarities in vaccination and natural exposures to specific immunogens—the former is simply a controlled variant of the latter. For parents intending to take their children for SIV, information about medical eligibility for SIV, vaccination clinic and costs and how to arrange, particularly the timing of the two vaccinations for children’s initial SIV, should be provided to enhance optimal timing of SIV.

Despite being ineffective for increasing children’s SIV uptake, the intervention was nonetheless highly acceptable for most participants. They appreciated the convenience of using WhatsApp messenger as a channel for health communication compared with sourcing information from websites or other traditional health communication methods. In addition, participants emphasized the importance of being able to interact with a health professional and thereby have access to more professional, trustworthy, and personalized information through WhatsApp. This indicates that the involvement of a health professional in the online communication is highly valued by parents and is likely to have greater impact if the health professional is a primary care provider to the target population. However, our study also indicates that audience segmentation, based on parents’ prior beliefs about SIV, is necessary for improving the effectiveness and acceptability of social networking interventions to achieve behavioral change. Putting people with different vaccination beliefs into one group may lead to strong arguments which may negatively affect other members’ participation in the discussion and the online communication environment. Finding approaches that work to bring resistant parents around to SIV requires further research.

### Limitations

This study had several limitations. First, we only recruited participants who were users of WhatsApp or those who were willing to install WhatsApp on their mobile phone and thereby the sample may not be representative for the target population, although the penetration rate of WhatsApp use was very high in the population. Since almost all participants reported using WhatsApp on a daily basis, the data did not have sufficient variance to allow for examining the intervention effects stratified by WhatsApp use. Second, a discussion group specifically for influenza vaccination may dissuade those uninterested in the topic, causing in-group biases. However, our analysis did not find significant differences in participants’ demographics, perceptions of SIV, and SIV history and intention across intervention arms. Third, this was a preliminary study to test social networking interventions effects on SIV uptake and as such the sample size was insufficient for detecting a small effect size. Fourth, data on children’s SIV uptake were reported by parents and could not be validated from children’s medical records and may be subject to social desirability bias. The survey was emphasized to be anonymous for participants to minimize social desirability bias and improve response rate. Fifth, in the WhatsApp discussion groups, out-of-office-hour discussions were not promptly monitored and addressed. The time lag in addressing participants’ questions or concerns may have affected participants’ subsequent participation in discussions and thereby SIV decision making. However, it is difficult to determine optimal moderator input in the WhatsApp discussion given the discussion group tried to encourage mutual support between participants. Furthermore, the infrequent emotional exchange among participants also indicated insufficient development of attachment to and friendships between group members, which could be a reason for why around half of the participants were lurkers, silent and passive members in the WhatsApp discussion. This represents to be a big challenge for the sustainability of online discussion. Future studies need to examine how to encourage information support from peers, moderate their emotional interactions, and the optimized moderator participation.

### Conclusion

The social networking intervention for mothers was ineffective for increasing SIV uptake among young children but did effectively increase mothers’ perceived self-efficacy for taking their children for SIV. A combination of social networking intervention with added time pressure on decision making can significantly promote children’s SIV among non–full-time working mothers, but among mothers working full-time, time pressure may reduce SIV uptake by reinforcing the influence of negative cues on SIV decision making. Future social networking interventions should consider audience segmentation using mothers’ working status and their prior SIV attitudes. Mothers’ participation in the online discussion mainly involved sharing concerns or negative views about vaccine safety, side effects, and effectiveness and seeking information or opinions to clarify these concerns. Mothers’ knowledge sharing and information giving was mainly supportive of those intending to take their children for SIV but seldom addressed concerns about vaccine safety, side effects, and effectiveness, possibly due to uncertainty around knowledge and information. The moderator played an important role by providing knowledge and information that addressed vaccine-related concerns and shaped positive online discussions about vaccination. Finally, our study indicates that WhatsApp messenger is a highly acceptable medium for health communication among parents in Hong Kong, but health professionals should be involved for more effective health communications.

## Appendix

Multimedia Appendix 1 Vaccination reminders (English and Chinese versions).

Multimedia Appendix 2 Guideline for monitoring and facilitating WhatsApp discussion for the moderator.

Multimedia Appendix 3 Supplementary tables.

Multimedia Appendix 4 Change of child's seasonal influenza vaccination uptake by intervention condition and participants' work status: (A) mothers with a full-time job and (B) mothers without a full-time job.

Multimedia Appendix 5 CONSORT EHEALTH checklist (V 1.6.1.).

## Abbreviations

CIVSS: Childhood Influenza Vaccination Subsidy Scheme
GEE: generalized estimating equation
SIV: seasonal influenza vaccination
SMS: short message service
SNI+TP: group that received weekly reminders to take their children for SIV via WhatsApp discussion groups with a time pressure component
SNI–TP: group that received weekly reminders to take their children for SIV via WhatsApp discussion groups without a time pressure component
