# Supplementary figures and images for: Effectiveness and Parental Acceptability of Social Networking Interventions for Promoting Seasonal Influenza Vaccination Among Young Children: Randomized Controlled Trial
Source: J Med Internet Res. 2020 Feb 28;22(2):e16427. doi: 10.2196/16427 (PMC7070348; doi:10.2196/16427)

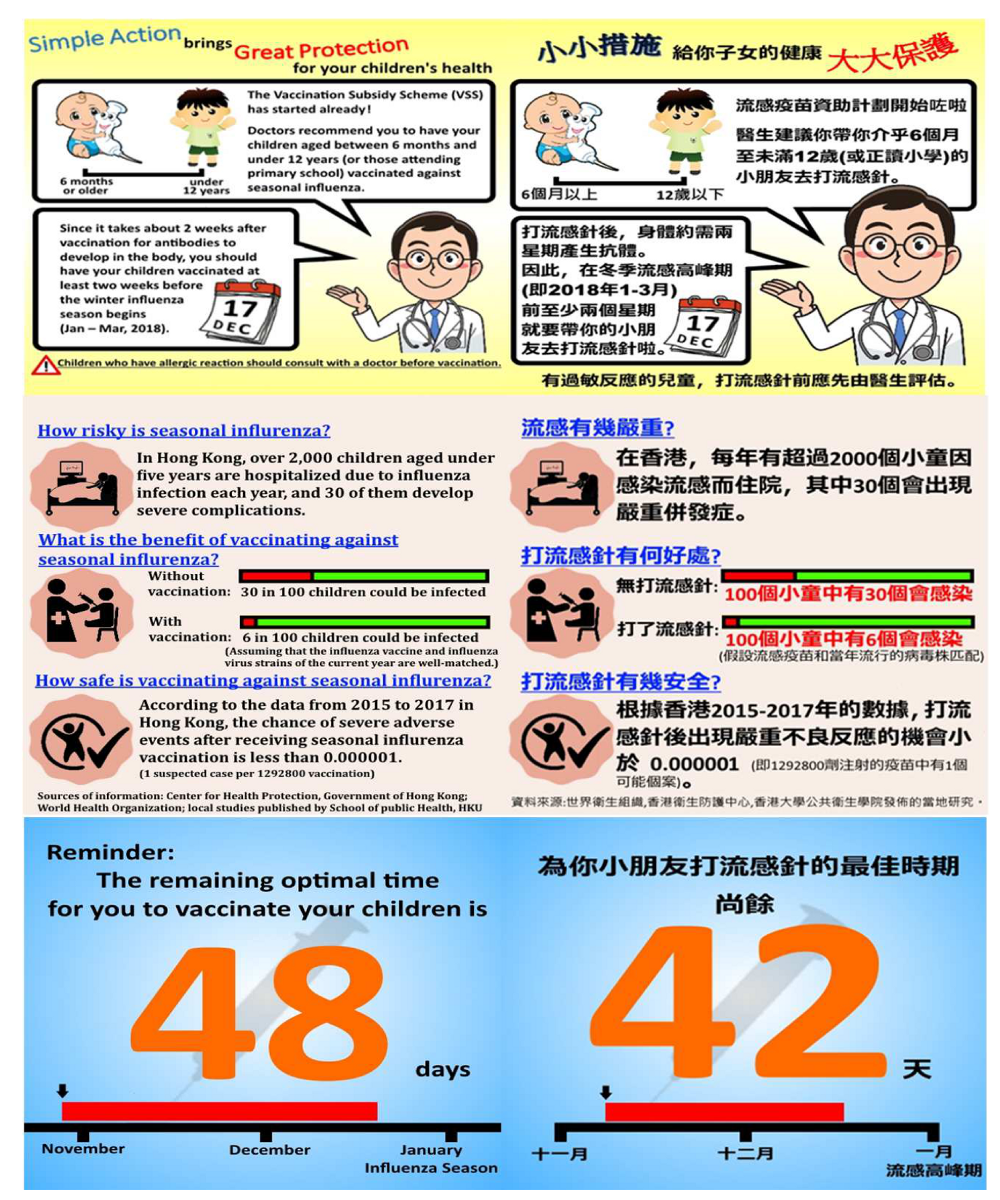

Supplement: Multimedia Appendix 1 [file jmir_v22i2e16427_app1.png]

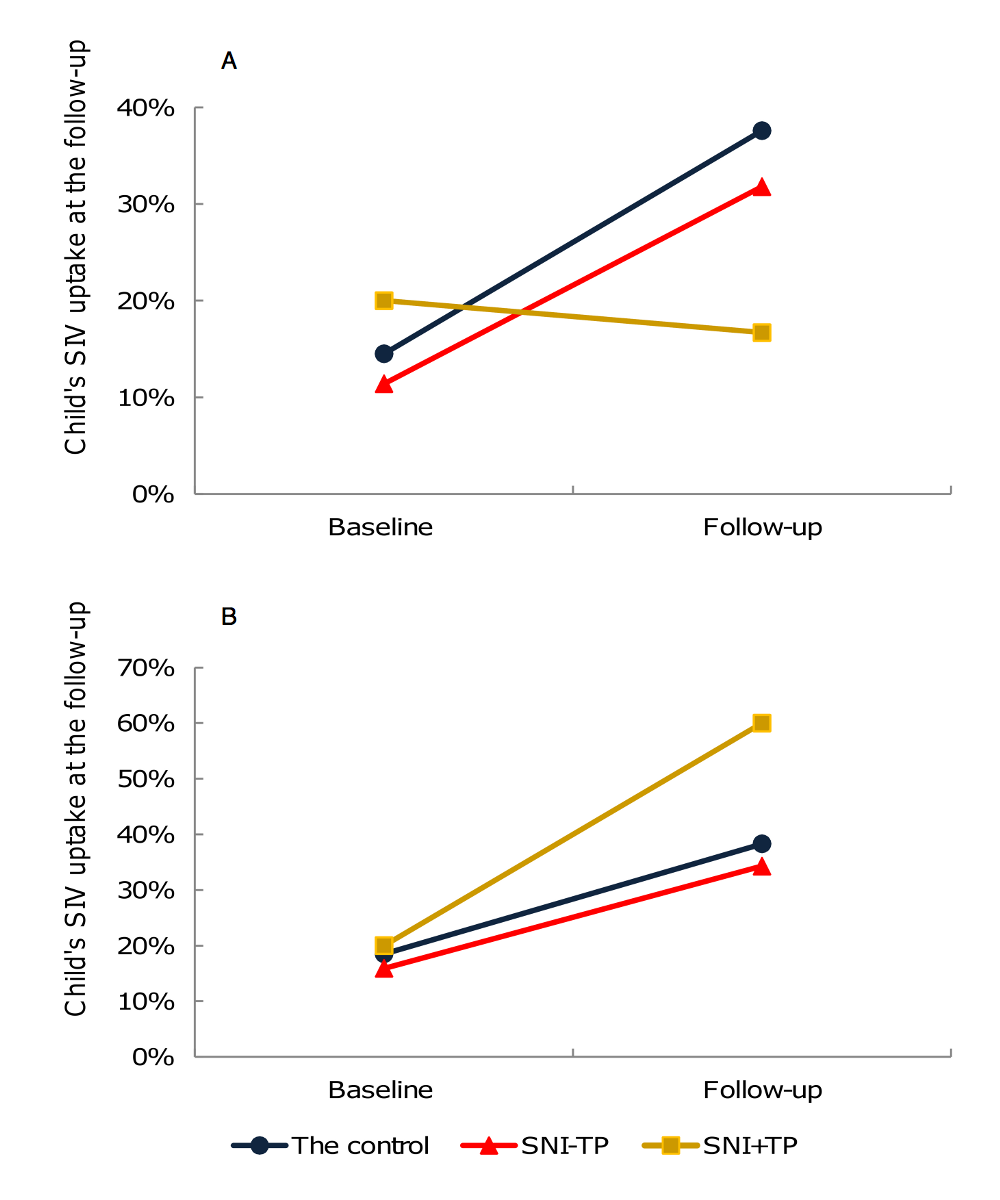

Supplement: Multimedia Appendix 4 [file jmir_v22i2e16427_app4.png]
