# Supplementary material for: Effectiveness and Parental Acceptability of Social Networking Interventions for Promoting Seasonal Influenza Vaccination Among Young Children: Randomized Controlled Trial
Source: J Med Internet Res. 2020 Feb 28;22(2):e16427. doi: 10.2196/16427 (PMC7070348; doi:10.2196/16427)
Supplement: Multimedia Appendix 2 [file jmir_v22i2e16427_app2.pdf]

Using social networking service to encourage parents to take their children for seasonal influenza vaccination

Moderator's guideline

1. Routine tasks moderators need to do

- Post the vaccination reminders once per week over the intervention periods (October-December).
- Encourage update of influenza vaccination uptake among the group members once a week (Appendix Table)
- Answer or clarify participants' questions, concerns and misunderstandings about influenza and influenza vaccination if these questions, concerns and misunderstandings are not addressed by other peers in the group. Responses to participants' questions, concerns and misunderstanding are constructed based on the FAQs set by CHP, CDC and WHO.
- Discourage posting of contents irrelevant to influenza and influenza vaccination in the group.
- Copy and send the conversation contents of the discussion groups to the PI every week.
- Remove all participants in the social group within two weeks after the intervention period ends.

2. How to enhance the positive influence of social norms

- Initiate the conversation by "according to our survey, the majority of mothers in our group intend to take their eligible children for seasonal influenza vaccination. Mothers who intend to do this should plan to get your children's vaccination as early as possible" (encouraging planning for vaccination) at the first week of the intervention.
- In subsequent weeks of the intervention, initiate the conversation by "Did any mothers take your child for seasonal influenza vaccination last week?" or "Any mothers plan to take your children for seasonal influenza vaccination in the coming week?"
- If no mothers respond to the above posts, influenza vaccination statistics among the target children in Hong Kong over the past one week will be posted.
- Posts are mainly aimed to enhance the positive influence of social norms, enhance social support in decision making. Additional posts that target components of the extended theory of planned behaviours will also be used (Appendix Table)

3. How to deal with the group members with inappropriate actions in the groups

- Discourage posting of contents irrelevant to influenza and influenza vaccination in the group and remind the participants to post relevant contents.
- Advise the group members to ignore the irrelevant posts.
- Set up participation rules and inform participants that if they seriously violate the participant rules, they will be expelled from the discussion group.

4. Other Rules

- Be the role model for the group and should not post offensive statement.
- Respect all group members and act in a manner that is considered polite, friendly, helpful and unbiased.
- Learn and be familiar with all of the regulations.
- Keep all the posts in the group as confidential and should not share and discuss the information in the group to others except the PI and co-investigators.
- Notify that group members have the right to withdraw from the social group. If any group member withdraws, moderator have to enquire the reasons and report the withdraw issue to the PI.

Appendix Table Examples of posts for encouraging influenza vaccination used in the WhatsApp discussion groups

Theoretical constructs	Objectives	Examples of posts

Social norms and action planning	To enhance the influence of positive social norms on children's SIV; To encourage planning for children's vaccination	"*According to our survey, most mothers indicated that they intent to take their child for flu vaccination. Mothers who have this intention are advised to plan the vaccination as early as possible.*"<br><br>"*Do any mothers plan to take your child for flu vaccination in the coming week?*"<br><br>"*Do any of your doctors, friends or family advise that you should take your child for flu vaccination?*"
Child's risk of infection	To enhance social support and promote mothers' perceived control over taking their child for SIV	"*Did any mothers take your child for flu vaccination over the past one week? We welcome mothers who had taken your child for flu vaccination to share your experience.*"<br><br>"*It is going to be the weekend. I know that many mothers have jobs during weekdays. Does anyone know which clinics provide vaccination services during weekends?*"<br><br>"*For mothers who intend to take your child for flu vaccination, we provide the following website for you. Through the website, you can search a clinic of your district that provides vaccination service. Information about the cost of vaccination services is also available on the website.*"<br><br>"*The winter flu season is coming, we suggest that mothers who haven't made a decision for whether to take your child for flu vaccination can discuss with each other or consult mothers wo had taken their child for flu vaccination to make a choice that is suitable for your child.*"<br><br>"*As far as I know, appointments of some clinics that provide free flu vaccination for children have been full. If mothers know any available clinics that provide vaccination services at a low cost can share with each other.*"
Risk of influenza to children	To increase mothers' perceived risk of children's susceptibility to influenza infection	"*The weather is becoming cold recently. There are increasing children seeking healthcare in private clinics due to respiratory infections. As the atmospheric temperature declines, activity of many respiratory viruses increases. Mothers and children are advised to pay attention to personal hygiene such as washing hands more frequently to minimize the transmission of viruses.*"
Benefit of influenza Vaccination		"*For influenza prevention, although handwashing is an effective preventive measure, influenza vaccination is considered to be more effective based on the conclusion of existing research findings.*"
Safety of influenza vaccination		"*According to the statistics from Hong Kong Center for Health Protection, since the 2017 CIVSS, a total of 160,700 doses of flu vaccine have been administered to the general public including adults and children. Of the 160,700 doses of flu vaccine administered, there was no report of adverse events following immunization. You may read more details about the statistics from the following website*" http://www.chp.gov.hk/en/view_content/26734.html
