# Supplementary material for: Effectiveness and Parental Acceptability of Social Networking Interventions for Promoting Seasonal Influenza Vaccination Among Young Children: Randomized Controlled Trial
Source: J Med Internet Res. 2020 Feb 28;22(2):e16427. doi: 10.2196/16427 (PMC7070348; doi:10.2196/16427)
Supplement: Multimedia Appendix 3 [file jmir_v22i2e16427_app3.docx]

# Multimedia Appendix 3. Supplementary tables.

Table A. Comparison of demographics, the target child’s characteristics, and history of SIV, baseline perceptions regarding childhood SIV and intention to take the target child for SIV among participants who completed the baseline and outcome assessments.

|  | Participants who completed the baseline | | | Participants who completed the outcome assessment | | |  |
| --- | --- | --- | --- | --- | --- | --- | --- |
|  | The Control (N=205) | SNI-TP (N=80) | SNI+TP (N=80) | The Control (N=174) | SNI-TP (N=57) | SNI+TP (N=60) | Difference (*P*-value) ^a^ |
| Age group (years) |  |  |  |  |  |  |  |
| 18-34 | 57.3% | 51.2% | 58.7% | 57.5% | 49.1% | 60.0% | .57 (.44) |
| ≥35 | 42.6% | 48.7% | 41.2% | 42.5% | 50.9% | 40.0% |  |
| Birth places |  |  |  |  |  |  |  |
| Hong Kong | 84.4% | 88.7% | 83.7% | 86.2% | 86.0% | 86.7% | .62 (.99) |
| Other places | 15.1% | 11.2% | 16.2% | 13.8% | 14.0% | 13.3% |  |
| Educational attainment |  |  |  |  |  |  |  |
| Secondary or below | 41.0% | 40.0% | 45.0% | 40.2% | 42.1% | 50.0% | .78 (.42) |
| Tertiary or above | 59.0% | 60.0% | 55.0% | 59.8% | 57.9% | 50.0% |  |
| Working status |  |  |  |  |  |  |  |
| Full-time | 54.1% | 45.0% | 50.0% | 53.4% | 38.6% | 50.0% | .37 (.15) |
| Part-time/unemployed | 45.8% | 55.0% | 50.0% | 46.5% | 61.4% | 50.0% |  |
| Income (HK$)^b^ |  |  |  |  |  |  |  |
| <20,000 | 12.7% | 6.2% | 10.0% | 11.5% | 8.8% | 8.3% | .46 (0.71) |
| 20,000-40,000 | 39.2% | 36.2% | 36.2% | 40.0% | 35.1% | 33.3% |  |
| ≥40,000 | 48.0% | 57.5% | 53.7% | 48.8% | 56.1% | 58.3% |  |
| Number of children |  |  |  |  |  |  |  |
| One | 81.9% | 80.0% | 76.2% | 83.3% | 78.9% | 78.3% | .55 (.60) |
| Two or more | 18.0% | 20.0% | 23.7% | 16.7% | 21.0% | 21.7% |  |
| Age of the youngest target child (months) |  |  |  |  |  |  |  |
| 6-12 | 19.5% | 26.2% | 17.5% | 17.8% | 24.6% | 16.7% | .40 (.55) |
| 12-36 | 45.4% | 35.0% | 48.7% | 47.1% | 35.1% | 45.0% |  |
| 36-72 | 35.1% | 38.7% | 33.7% | 35.1% | 40.3% | 38.3% |  |
| Gender of the youngest target child |  |  |  |  |  |  |  |
| Girl | 49.8% | 38.7% | 46.2% | 49.4% | 38.6% | 46.7% | .25 (.36) |
| Boy | 50.2% | 61.2% | 53.7% | 50.6% | 61.4% | 53.3% |  |
| Participants’ past-12-months SIV uptake (yes) | 12.2% | 12.5% | 12.5% | 13.2% | 14.0% | 15.0% | .99 (.94) |
| Youngest target child’s past-12-months SIV uptake (yes) | 16.3% | 13.9% | 20.0% | 18.0% | 16.1% | 16.7% | .58 (.93) |
| Intention to take child for SIV |  |  |  |  |  |  |  |
| Very unlikely/unlikely | 21.9% | 22.5% | 17.5% | 22.4% | 22.8% | 16.7% | .17 (.35) |
| Evens | 38.5% | 38.7% | 27.5% | 39.7% | 36.8% | 30.0% |  |
| Likely/very likely | 39.5% | 38.7% | 55.0% | 37.9% | 40.3% | 53.3% |  |
| Worry about child being infected with flu ^c^ | 46.8% | 42.5% | 48.7% | 48.3% | 42.1% | 50.0% | .71 (.65) |
| Believing that SIV can reduce risk of infection for children ^c^ | 59.0% | 52.5% | 60.0% | 58.6% | 54.4% | 61.7% | .55 (.72) |
| Feeling confident to take child for SIV ^c^ | 66.3% | 63.7% | 61.2% | 67.2% | 63.2% | 60.0% | .71 (.57) |
| Believing that SIV is safe for my child ^c^ | 49.3% | 46.2% | 42.5% | 51.7% | 47.4% | 41.7% | .58 (.39) |
| Frequency of using WhatsApp |  |  |  |  |  |  |  |
| About hourly or more frequently | 95.6% | 96.2% | 95.0% | 97.1% | 96.5% | 98.3% | - |
| 4-6 times a day | 3.4% | 3.7% | 1.2% | 2.3% | 3.5% | 1.7% |  |
| 1-3 times a day | 1.0% | 0 | 2.5% | 0.6% | 0 | 0 |  |
| Less frequent than daily | 0 | 0 | 1.2% |  |  |  |  |

^a^ P-values were calculated based on Pearson chi-square test; The P-values outside of the brackets were for the comparisons of participants who completed the baseline assessment while those inside of the brackets were for the comparison of participants who completed the outcome assessment;

^b^ 1HK$=0.13 US$

^c^ Responses of these items were coded as “1=agree/strong agree” and “0=strongly disagree/disagree/evens”;

SNI-TP: social-networking intervention group who received weekly vaccination reminders without time pressure component; SNI+TP: social-networking intervention group who received weekly vaccination reminders with time pressure component; SIV: seasonal influenza vaccination.

Table B. Assessment of the intervention effects on parental perceptions using generalized estimating equations logistic regression.

|  | Beta (SE) | Odds ratios (95% CI) | *P*-value |
| --- | --- | --- | --- |
| **Model1: Worry about child being infected with flu** |  |  |  |
| Intervention: |  |  |  |
| SNI-TP (vs. Control) | -.18 (0.27) | 0.84 (0.50-1.41) | .51 |
| SNI+TP (vs. Control) | .08 (0.26) | 1.08 (0.64-1.81) | .77 |
| Time effect: follow-up vs. baseline | .06 (0.19) | 1.06 (0.73-1.53) | .76 |
| Time x SNI-TP | -.07 (0.38) | 0.93 (0.44-1.96) | .84 |
| Time x SNI+TP | -.07 (0.31) | 0.93 (0.51-1.70) | .81 |
| **Model 2: Perceived benefit of SIV for children** |  |  |  |
| Intervention: |  |  |  |
| SNI-TP (vs. Control) | -.26 (0.27) | 0.77 (0.46-1.29) | .32 |
| SNI+TP (vs. Control) | .04 (0.27) | 1.04 (0.61-1.76) | .88 |
| Time effect: follow-up vs. baseline | -.02 (0.17) | 0.98 (0.70-1.38) | .92 |
| Time x SNI-TP | .38 (0.36) | 1.46 (0.72-2.98) | .29 |
| Time x SNI+TP | .54 (0.37) | 1.71 (0.84-3.51) | .14 |
| **Model 3: Perceived safety of SIV for children** |  |  |  |
| Intervention: |  |  |  |
| SNI-TP (vs. Control) | -.12 (0.27) | 0.89 (0.53-1.49) | .65 |
| SNI+TP (vs. Control) | -.27 (0.27) | 0.76 (0.45-1.28) | .30 |
| Time effect: follow-up vs. baseline | .24 (0.17) | 1.27 (0.91-1.77) | .16 |
| Time x SNI-TP | -.02 (0.37) | 1.02 (0.49-2.11) | .96 |
| Time x SNI+TP | .27 (0.32) | 1.30 (0.69-2.46) | .41 |
| **Model 4: Perceived self-efficacy for taking children for SIV** |  |  |  |
| Intervention: |  |  |  |
| SNI-TP (vs. Control) | -.11 (0.27) | 0.89 (0.52-1.53) | .68 |
| SNI+TP (vs. Control) | -.22 (0.27) | 0.80 (0.47-1.37) | .42 |
| Time effect: follow-up vs. baseline | .12 (0.18) | 1.13 (0.79-1.60) | .50 |
| Time x SNI-TP | .99 (0.47) | 2.69 (1.07-6.79) | .036 |
| Time x SNI+TP | .92 (0.41) | 2.50 (1.13-5.55) | .024 |

Table C. Characteristics of the posts from participants and the moderator in the WhatsApp discussion groups.

|  | Total | SNI-TP1 | SNI-TP2 | SNI+TP1 | SNI+TP2 |
| --- | --- | --- | --- | --- | --- |
| **Participants’ posts** |  |  |  |  |  |
| Frequency of post distribution |  |  |  |  |  |
| Zero | 41.9% | 55.0% | 40.0% | 42.5% | 30.0% |
| 1-4 | 34.4% | 27.5% | 30.0% | 35.0% | 45.0% |
| 5-9 | 15.6% | 10.0% | 22.5% | 12.5% | 17.5% |
| 10 or more | 8.1% | 7.5% | 7.5% | 10.0% | 7.5% |
| Median number of post/participants (range) | 1 (0-51) | 0 (0-17) | 1 (0-21) | 1 (0-51) | 1 (0-17) |
| Number of post/participant (SD) | 3.08 (5.90) | 2.20(4.05) | 3.25 (4.63) | 4.22 (9.37) | 2.65 (3.72) |
| Total relevant posts ^a^ | 434 | 90 | 114 | 135 | 95 |
| Textual | 398 | 67 | 111 | 130 | 90 |
| Graphical | 7 | 6 | 1 | 0 | 0 |
| Hyperlink | 7 | 1 | 2 | 1 | 3 |
| Emoji | 57 | 17 | 12 | 18 | 10 |
| **Moderators’ post** |  |  |  |  |  |
| Vaccination reminders | 32 | 8 | 8 | 8 | 8 |
| Other posts | 203 | 42 | 64 | 51 | 46 |
| Textual posts | 190 | 37 | 60 | 49 | 44 |
| Graphical posts | 4 | 1 | 1 | 1 | 1 |
| Hyperlinks | 10 | 2 | 3 | 3 | 2 |
| Emoji | 6 | 4 | 2 | 0 | 0 |

^a^ One post can contains different formats; SD: standard deviation

SNI-TP1 and SNI-TP2 were the two WhatsApp discussion groups received vaccination reminders without time pressure component while SNI+TP1 and SNI+TP2 were the two WhatsApp discussion groups received vaccination reminders with time pressure component.

^b^ Percentage of posts that were made during weekend or out of office hours (9:00am-6:00pm) during weekdays of the total relevant posts across discussion groups.

Table D. Major themes and quotes from the qualitative interview on parental acceptability for the intervention using the WhatsApp discussion groups.

| Themes and categories | Examples of quotes |
| --- | --- |
| **Perceptions of information from the moderator** |  |
| Information attributes |  |
| Positive attributes | - *… The moderator explained very clear. The explanations are what we want to know. I found he answered others’ questions very clearly.* - *I think the moderator did a good job because when he can answer my questions and after reading his explanations…I think he can explain other mothers' and my concerns one by one…I mean for those I feel uncertain, he can explain the details.* |
| Negative attributes | - *Initially, it (the reminder) made me to think whether I should take my child for flu vaccination…yes…but later….I started to feel that …it seemed to repeat too frequently.* |
| Benefits of information provision |  |
| Knowledge acquisition | - *(It is useful) because it provides some data for analysis and discussion, and let me think whether I really need it (vaccination). Some information is new for me.* - *At least it let me know when will be the influenza season* |
| Moving to a contemplation stage | - *It motivated me to think whether I should take my child for flu vaccination or not* - *"Because…what he said is …how to say…it seems to be a reminder…a kindly reminder for you that….it is time to take your child for flu vaccination or the flu vaccine…er….it is useful for protecting against flu but it is not to force you to do that…or not that you must do this...you can read it...so ...reading is reading...but finally you need to make the decision yourself"* |
| Promoting motivation for influenza vaccination | - *Before participating in the discussion group…er…possibly I did not have such a strong motivation to take my child for flu vaccination…I should say that.* - *Yes, I mean through the communication, during that period…that period is not short, although I did not ask question and participate in the discussion, I can read the information from the group and it changed my though about it.* - *Because first...actually I had already encountered all those discussed in the group from other online sources. I felt that …I mean I felt that I had already seen the information provided by the group moderator. I felt that the information is the same as what I had already searched and seen from the internet. In addition…I remembered I seemed to have asked one or two questions. I mean that I initially had some concerns over flu vaccination, but then after asking questions...after he provided me some information I need, I felt that the information is correct. So the information helped me to make the decision.* |
| Reminding vaccination planning | - *It is quite useful. Because we sometimes…possibly did not pay too much attention to it…I mean for this issue (flu vaccination), and we also would not think about too much details about it such as where we can get it or when to take our child for flu vaccination, and something like that.* - *I just participated in the discussion group to know when the vaccine was available so that I can book the appointment for my child’s vaccination.* |
| Lack of interest in the information | - *...It is just a reminder because I have made arrangement for my two children to receive flu vaccination. So your reminder did not have a lot of impact on my decision. I had already book a time for my children's flu vaccination* - *Actually there should be similar information from the internet. No need to share the information with others"* |
| **Perceived advantages of using WhatsApp for promoting children’s health** |  |
| Information accessibility | - *I think it is actually good to use WhatsApp, because the information technology is advanced nowadays…you don't need to ask people to come out and waste their time…they can read when they have time….because some mothers who take care of children….several mothers I know….they are really busy and possibly can only have time to read the information at night...till the end of a day.* |
| Information quality | - *The information is more in-depth and is different from general information. Also people can share their own experience or what they know, and different kinds of information.* |
| Interaction with a health professional | - *Because actually it is good…I mean…if you search the information from the internet…you may not be able to ask questions but we can ask question in the WhatsApp and the answers are easier to be understood* |
| **Concerns over using WhatsApp for health promotion** | - *I have no concern (for participating in this group). Instead, I would concern over participating in some discussions groups that are…because I would not…I don’t think it (disclosure of mobile phone number) can be a concern…instead…I would concern for participating in those discussion groups that are…aimed for selling something. This is my main concern and I won’t participate in those discussion groups.* |
| **Contributors of not participating in the online discussion** |  |
| Perceived low confidence about giving information | - *(I didn’t share information)…on the one hand I don’t know too much about it…and the moderator should be able to give a clearer explanation* - *Because there is already health professional there. I don't want to be challenged by others."* |
| Avoiding arguments | - *…I did not ask questions…because by the end the whole group …Possibly because there were too many people in the group and they have different opinions. Some of them seemed to support and others seemed to disagree (with flu vaccination). So I usually did not answer but only read ...I felt that I should not say too much....I just received the information...I don't want them to argue for this. This is not good* |
| Perceived low information need | - *Actually, I did not ask questions. Because I found the explanations have already been very clear in the discussion group. I found the moderator explained very clear and… other mothers had asked what I want to know. So I did not ask questions myself"* - *"Yes, (it is useful). Some information is difficult….you don't know where to get this information…excepting for asking a doctor…but the doctors are always too busy to give you explanations. You can ask the more professional persons by participating in such WhatsApp discussion groups* |
| **Perceptions of the time pressure component** | - *"It makes me to think that I should make a rapid decision, to take or not to take my child for flu vaccination.* |
